# Hexane-1,6-diammonium dinitrate

**DOI:** 10.1107/S1600536809012963

**Published:** 2009-04-08

**Authors:** Charmaine van Blerk, Gert J. Kruger

**Affiliations:** a University of Johannesburg, Department of Chemistry, P O Box 524, Auckland Park, Johannesburg 2006, South Africa

## Abstract

The hexane-1,6-diammonium cation of the title compound, C_6_H_18_N_2_^2+^·2NO_3_^−^, lies across a crystallographic inversion centre and shows significant deviation from planarity in the hydro­carbon chain. This is evident from the torsion angle of −64.0°(2) along the N—C—C—C bond and thse torsion angle of −67.1°(2) along the C—C—C—C bonds. An intricate three-dimensional hydrogen-bonding network exists in the crystal structure, with each H atom on the ammonium group exhibiting bifurcated inter­actions to the nitrate anion. Complex hydrogen-bonded ring and chain motifs are also evident, in particular a 26-membered ring with graph-set notation *R*_4_^4^(26) is observed.

## Related literature

For related structural studies of hexane-1,6-diammonium salts, see: van Blerk & Kruger (2008 ▶). For hydrogen-bond motifs, see: Bernstein *et al.* (1995 ▶). For a description of the Cambridge Structural Database, see: Allen (2002 ▶). 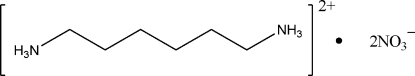


## Experimental

### 

#### Crystal data

C_6_H_18_N_2_^2+^·2NO_3_^−^
*M* *_r_* = 242.24
Monoclinic, 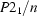

*a* = 6.2947 (1) Å
*b* = 11.6783 (3) Å
*c* = 8.1211 (2) Å
β = 92.840 (1)°
*V* = 596.26 (2) Å^3^
*Z* = 2
Mo *K*α radiation
μ = 0.12 mm^−1^
*T* = 295 K
0.46 × 0.20 × 0.16 mm

#### Data collection

Bruker SMART CCD diffractometer
Absorption correction: multi-scan (AX-Scale; Bruker, 2008 ▶) *T*_min_ = 0.948, *T*_max_ = 0.981
14665 measured reflections
1718 independent reflections
1107 reflections with *I* > 2σ(*I*)
*R*_int_ = 0.026

#### Refinement

*R*[*F* ^2^ > 2σ(*F* ^2^)] = 0.045
*wR*(*F* ^2^) = 0.147
*S* = 1.02
1718 reflections
74 parameters
H-atom parameters constrained
Δρ_max_ = 0.37 e Å^−3^
Δρ_min_ = −0.19 e Å^−3^

### 

Data collection: *SMART-NT* (Bruker, 1998 ▶); cell refinement: *SAINT* (Bruker, 2008 ▶); data reduction: *SAINT*; program(s) used to solve structure: *SHELXS97* (Sheldrick, 2008 ▶); program(s) used to refine structure: *SHELXL97* (Sheldrick, 2008 ▶); molecular graphics: *X-SEED* (Barbour, 2001 ▶) and *Mercury* (Macrae *et al*., 2006 ▶); software used to prepare material for publication: *publCIF* (Westrip, 2009 ▶).

## Supplementary Material

Crystal structure: contains datablocks I, global. DOI: 10.1107/S1600536809012963/fj2206sup1.cif

Structure factors: contains datablocks I. DOI: 10.1107/S1600536809012963/fj2206Isup2.hkl

Additional supplementary materials:  crystallographic information; 3D view; checkCIF report

## Figures and Tables

**Table 1:** Hydrogen-bond geometry (Å, °)

*D*—H⋯*A*	*D*—H	H⋯*A*	*D*⋯*A*	*D*—H⋯*A*
N1—H1*C*⋯O2	0.89	2.53	3.1760 (19)	130
N1—H1*C*⋯O3	0.89	2.04	2.9184 (19)	171
N1—H1*D*⋯O1^ii^	0.89	2.13	2.9692 (18)	158
N1—H1*D*⋯O2^ii^	0.89	2.36	3.1374 (19)	146
N1—H1*E*⋯O1^iii^	0.89	2.26	3.0561 (18)	149
N1—H1*E*⋯O3^iii^	0.89	2.23	3.0110 (18)	146

Symmetry codes: (ii) 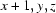; (iii) 
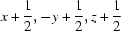
.

**Fig. 1.**
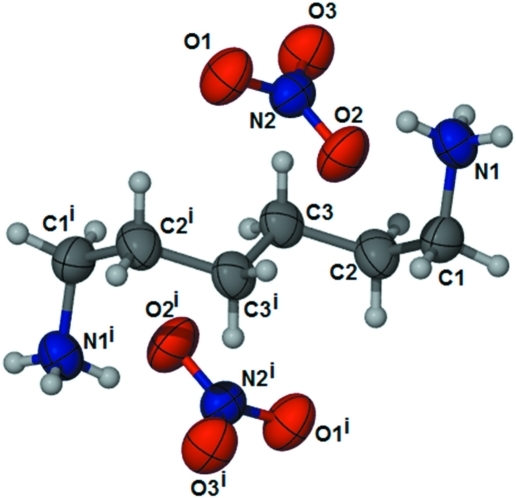
: Molecular structure of (I), with atomic numbering scheme and displacement ellipsoids drawn at the 50% probability level. Atoms labelled with (i) are at the symmetry position (1 - x, - y, - z)

**Fig. 2.**
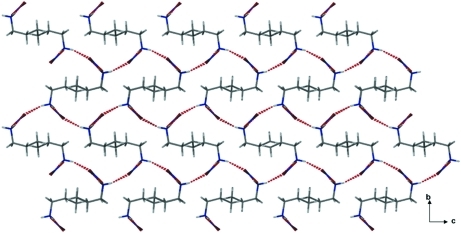
: Packing arrangement if (I) viewed down the a axis. Hydrogen bonds are indicated by dashed lines.

**Fig. 3.**
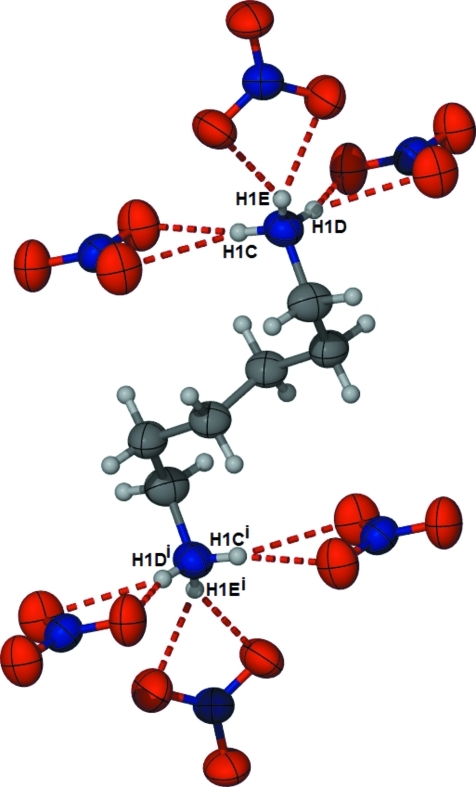
: Close-up view of (I) viewed down the a axis clearly showing the bifurcated hydrogen-bonding interactions. Hydrogen bonds are indicated by dashed lines.

**Fig. 4.**
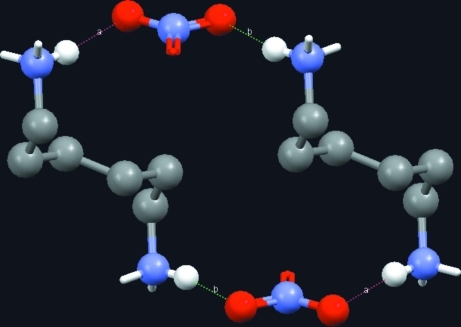
: Close up view of (I) viewed down the c axis showing the ring motif involving two diammonium cations and two nitrate anions. Hydrogen atoms on the hydrocarbon chain are omitted for clarity.

## supplementary crystallographic information

### Comment

The crystal structure of the title compound (I) adds to our current ongoing studies of long-chained diammonium salts. Colourless needle-like rectangular crystals of hexane-1,6-diammonium dinitrate were synthesized and formed as part of our structural chemistry study of the inorganic mineral acid salts of hexane-1,6-diamine. A search of the Cambridge Structural Database (Version 5.30, February 2009 release; Allen, 2002) revealed that this compound had not previously been determined.

The diammonium hexane chain lies across a crystallographic inversion centre and hence the asymmetric unit contains one nitrate anion and one-half of the hexane diammonium cation. The hydrocarbon chain is also not extended as is common in long chained hydrocarbons but shows significant folding and deviation from planarity. This is clearly evident from the torsion angle along the N1—C1—C2—C3 bond (–64.0°(2)) and along the C1—C2—C3—C3^i^ bond (–67.1°(2)). Selected torsion angles can be found in Table 1. The molecular structure of (I) is shown in Figure 1.

Figure 2 illustrates the layered packing arrangement of the title compound (I). Single stacked layers of folded cations pack in between layers of nitrate anions showing a distinct inorganic - organic layering effect that is a common feature of these long-chained diammonium salts. The diammonium cations form bridges between the nitrate anion layers and an extensive three-dimensional hydrogen-bonding network is formed.

A close-up view of the hydrogen bonding interactions can be viewed in Figure 3 where very clear evidence of bifurcated interactions can be seen on each hydrogen atom of both ammonium groups. The hydrogen bond distances and angles for (I) can be found in Table 2. Since the hydrogen bonding network is complex, we focus on one particularly interesting hydrogen-bonding ring motif in the structure. Figure 4 shows a view of two diammonium cations and two nitrate anions (viewed down the *c* axis) that are hydrogen bonded together to form a large, 26-membered ring motif with graph set notation *R*_4_^4^(26). Another smaller ring motif is evident as a result of the bifurcated hydrogen-bond interaction with the nitrate anion and this ring has the graph-set notation *R*^2^_1_(4) but is not depicted graphically. Chain motifs also exist and were identified with *Mercury* (Macrae *et al.*), but again, these are not shown graphically.

### Experimental

Compound (I) was prepared by adding 1,6-diamino-hexane (0.50 g, 4.30 mmol) to 55% nitric acid (2 ml, 42.5 mmol) in a sample vial. The mixture was then refluxed at 363 K for 2 h. The solution was cooled at 2 K h^-1^ to room temperature. Colourless rectangular needles of hexane-1,6-diammonium dinitrate were collected and a suitable single-crystal was selected for the X-ray diffraction study.

### Refinement

H atoms were geometrically positioned and refined in the riding-model approximation, with C—H = 0.97 Å, N—H = 0.89 Å, and *U*_iso_(H) = 1.2Ueq(C) or 1.5Ueq(N). For (I), the highest peak in the final difference map is 0.99Å from C3 and the deepest hole is 0.63Å from N2.

### Crystal data

**Table:**

C_6_H_18_N_2_^2+^·2NO_3_^−^	*F*(000) = 260
*M**_r_* = 242.24	*D*_x_ = 1.349 Mg m^−^^3^
Monoclinic, *P*2_1_/*n*	Mo *K*α radiation, λ = 0.71073 Å
Hall symbol: -P 2yn	Cell parameters from 5933 reflections
*a* = 6.2947 (1) Å	θ = 2.5–25.2°
*b* = 11.6783 (3) Å	µ = 0.12 mm^−^^1^
*c* = 8.1211 (2) Å	*T* = 295 K
β = 92.840 (1)°	Rectangular, colourless
*V* = 596.26 (2) Å^3^	0.46 × 0.20 × 0.16 mm
*Z* = 2	

### Data collection

**Table:**

Bruker SMART CCD diffractometer	1718 independent reflections
Radiation source: fine-focus sealed tube	1107 reflections with *I* > 2σ(*I*)
graphite	*R*_int_ = 0.026
φ and ω scans	θ_max_ = 30.0°, θ_min_ = 3.1°
Absorption correction: multi-scan (AX-Scale; Bruker, 2008)	*h* = −8→8
*T*_min_ = 0.948, *T*_max_ = 0.981	*k* = −16→16
14665 measured reflections	*l* = −11→11

### Refinement

**Table:**

Refinement on *F*^2^	Primary atom site location: structure-invariant direct methods
Least-squares matrix: full	Secondary atom site location: difference Fourier map
*R*[*F*^2^ > 2σ(*F*^2^)] = 0.045	Hydrogen site location: inferred from neighbouring sites
*wR*(*F*^2^) = 0.147	H-atom parameters constrained
*S* = 1.02	*w* = 1/[σ^2^(*F*_o_^2^) + (0.0674*P*)^2^ + 0.1254*P*] where *P* = (*F*_o_^2^ + 2*F*_c_^2^)/3
1718 reflections	(Δ/σ)_max_ < 0.001
74 parameters	Δρ_max_ = 0.37 e Å^−^^3^
0 restraints	Δρ_min_ = −0.19 e Å^−^^3^

### Special details

**Table:**

Geometry. All e.s.d.'s (except the e.s.d. in the dihedral angle between two l.s. planes) are estimated using the full covariance matrix. The cell e.s.d.'s are taken into account individually in the estimation of e.s.d.'s in distances, angles and torsion angles; correlations between e.s.d.'s in cell parameters are only used when they are defined by crystal symmetry. An approximate (isotropic) treatment of cell e.s.d.'s is used for estimating e.s.d.'s involving l.s. planes.
Refinement. Refinement of *F*^2^ against ALL reflections. The weighted *R*-factor *wR* and goodness of fit *S* are based on *F*^2^, conventional *R*-factors *R* are based on *F*, with *F* set to zero for negative *F*^2^. The threshold expression of *F*^2^ > σ(*F*^2^) is used only for calculating *R*-factors(gt) *etc*. and is not relevant to the choice of reflections for refinement. *R*-factors based on *F*^2^ are statistically about twice as large as those based on *F*, and *R*- factors based on ALL data will be even larger.

### Fractional atomic coordinates and isotropic or equivalent isotropic displacement parameters (Å^2^)

**Table:**

	*x*	*y*	*z*	*U*_iso_*/*U*_eq_	
C1	0.7036 (3)	0.01369 (13)	0.31554 (19)	0.0599 (4)	
H1A	0.5614	−0.0134	0.3351	0.072*	
H1B	0.8020	−0.0243	0.3934	0.072*	
C2	0.7584 (3)	−0.01708 (14)	0.1427 (2)	0.0593 (4)	
H2A	0.7644	−0.0999	0.1351	0.071*	
H2B	0.9000	0.0117	0.1251	0.071*	
C3	0.6095 (2)	0.02648 (14)	0.00229 (18)	0.0540 (4)	
H3A	0.5955	0.1088	0.0126	0.065*	
H3B	0.6737	0.0110	−0.1016	0.065*	
N1	0.7138 (2)	0.13945 (11)	0.34353 (15)	0.0544 (4)	
H1C	0.6044	0.1730	0.2893	0.082*	
H1D	0.8350	0.1667	0.3072	0.082*	
H1E	0.7082	0.1539	0.4508	0.082*	
N2	0.2075 (2)	0.23209 (11)	0.22415 (16)	0.0510 (3)	
O1	0.03639 (19)	0.26482 (12)	0.15889 (16)	0.0710 (4)	
O2	0.2102 (2)	0.15918 (11)	0.33405 (15)	0.0732 (4)	
O3	0.3767 (2)	0.27276 (12)	0.17480 (16)	0.0714 (4)	

### Atomic displacement parameters (Å^2^)

**Table:**

	*U*^11^	*U*^22^	*U*^33^	*U*^12^	*U*^13^	*U*^23^
C1	0.0791 (11)	0.0552 (9)	0.0448 (8)	0.0006 (8)	−0.0042 (7)	0.0031 (6)
C2	0.0622 (9)	0.0591 (9)	0.0559 (9)	0.0067 (7)	−0.0052 (7)	−0.0109 (7)
C3	0.0632 (9)	0.0583 (9)	0.0407 (7)	−0.0024 (7)	0.0046 (6)	−0.0062 (6)
N1	0.0544 (8)	0.0610 (8)	0.0479 (7)	−0.0035 (6)	0.0033 (6)	−0.0087 (5)
N2	0.0584 (8)	0.0501 (7)	0.0451 (7)	0.0059 (6)	0.0070 (6)	−0.0002 (5)
O1	0.0567 (7)	0.0808 (9)	0.0755 (9)	0.0130 (6)	0.0018 (6)	0.0188 (6)
O2	0.0782 (9)	0.0798 (8)	0.0615 (7)	0.0012 (7)	0.0045 (6)	0.0279 (6)
O3	0.0576 (8)	0.0846 (9)	0.0731 (8)	−0.0041 (6)	0.0131 (6)	0.0188 (6)

### Geometric parameters (Å, °)

**Table:**

C1—N1	1.487 (2)	C3—H3A	0.9700
C1—C2	1.505 (2)	C3—H3B	0.9700
C1—H1A	0.9700	N1—H1C	0.8900
C1—H1B	0.9700	N1—H1D	0.8900
C2—C3	1.527 (2)	N1—H1E	0.8900
C2—H2A	0.9700	N2—O2	1.2330 (16)
C2—H2B	0.9700	N2—O1	1.2369 (17)
C3—C3^i^	1.510 (3)	N2—O3	1.2504 (17)
			
N1—C1—C2	111.60 (13)	C2—C3—H3A	108.7
N1—C1—H1A	109.3	C3^i^—C3—H3B	108.7
C2—C1—H1A	109.3	C2—C3—H3B	108.7
N1—C1—H1B	109.3	H3A—C3—H3B	107.6
C2—C1—H1B	109.3	C1—N1—H1C	109.5
H1A—C1—H1B	108.0	C1—N1—H1D	109.5
C1—C2—C3	117.17 (14)	H1C—N1—H1D	109.5
C1—C2—H2A	108.0	C1—N1—H1E	109.5
C3—C2—H2A	108.0	H1C—N1—H1E	109.5
C1—C2—H2B	108.0	H1D—N1—H1E	109.5
C3—C2—H2B	108.0	O2—N2—O1	120.27 (14)
H2A—C2—H2B	107.2	O2—N2—O3	120.85 (14)
C3^i^—C3—C2	114.07 (17)	O1—N2—O3	118.86 (13)
C3^i^—C3—H3A	108.7		
			
N1—C1—C2—C3	−64.0 (2)	C1—C2—C3—C3^i^	−67.1 (2)

Symmetry codes: (i) −*x*+1, −*y*, −*z*.

### Hydrogen-bond geometry (Å, °)

**Table:**

*D*—H···*A*	*D*—H	H···*A*	*D*···*A*	*D*—H···*A*
N1—H1C···O2	0.89	2.53	3.1760 (19)	130
N1—H1C···O3	0.89	2.04	2.9184 (19)	171
N1—H1D···O1^ii^	0.89	2.13	2.9692 (18)	158
N1—H1D···O2^ii^	0.89	2.36	3.1374 (19)	146
N1—H1E···O1^iii^	0.89	2.26	3.0561 (18)	149
N1—H1E···O3^iii^	0.89	2.23	3.0110 (18)	146

Symmetry codes: (ii) *x*+1, *y*, *z*; (iii) *x*+1/2, −*y*+1/2, *z*+1/2.
